# Assessment of Post-Resuscitation Intestinal Injury and Timing of Bacterial Translocation in Swine Anaesthetized With Propofol-Based Total Intravenous Anaesthesia

**DOI:** 10.7759/cureus.10362

**Published:** 2020-09-10

**Authors:** Andreas Tassopoulos, Athanasios Chalkias, Apostolos Papalois, Paraskevi Karlovasiti, Jacopo Sergio Antonio Zanda, Stefanos Chatzidakis, Maria Gazouli, Nicoletta Iacovidou, Daniela Fanni, Theodoros Xanthos

**Affiliations:** 1 Department of Cardiology, Red Cross General Hospital, Athens, GRC; 2 Department of Anesthesiology, University of Thessaly, School of Health Sciences, Faculty of Medicine, Larisa, GRC; 3 Translational Research and Training, ELPEN Research & Experimental Center, Athens, GRC; 4 Department of Biopathology - Microbiology and Biochemistry, Medical School, National and Kapodistrian University of Athens, Athens, GRC; 5 Division of Pathology, Department of Surgical Sciences, University of Cagliari, Cagliari, ITA; 6 Department of Medicine, European University Cyprus, School of Medicine, Nicosia, CYP; 7 Laboratory of Biology, Medical School, National and Kapodistrian University of Athens, Athens, GRC; 8 Department of Neonatology, Medical School, National and Kapodistrian University of Athens, Athens, GRC; 9 Division of Pathology, Department of Medical Sciences and Public Health, University of Cagliari, Cagliari, ITA; 10 Department of Physiology and Pathophysiology, European University Cyprus, School of Medicine, Nicosia, CYP

**Keywords:** bacterial translocation, propofol, cardiac arrest, post-resuscitation period, reperfusion

## Abstract

Introduction and objectives

Bacterial translocation (BT) is the passage of viable bacteria or endotoxins from the gastrointestinal lumen to extra-luminal tissues and is usually observed after intestinal ischaemia-reperfusion injury. The aim of this study was to investigate post-resuscitation BT after cardiac arrest and resuscitation in a swine anaesthetized with propofol-based total intravenous anaesthesia.

Materials and methods

Eighteen female Landrace/Large White piglets were randomly divided into control (CON), cardiac arrest (CA) and cardiac arrest-cardiopulmonary resuscitation (CA-CPR) groups. In the CON group, the animals were only monitored for two hours. In the CA group, the animals were not resuscitated and underwent necropsy immediately after cardiac arrest. In the CA-CPR group, the animals were resuscitated until the return of spontaneous circulation (ROSC) and were monitored for two hours. The animals of the CON and CA-CPR groups underwent necropsy 24 hours later. Bacterial translocation was assessed by blood and tissue cultures and endotoxin measurement in the portal and systemic circulation. Malondialdehyde content calculation and histological analysis of the intestine were performed in order to estimate ischemia and reperfusion (I/R) tissue damage.

Results

Malondialdehyde content, an indicator of oxidative stress, was significantly higher in the CA-CPR group compared to the CA in homogenized ileum (p=0.016). Malondialdehyde content in homogenized colon revealed significantly higher levels in the CA-CPR group compared to the CON (p=0.004) and the CA group (p=0.016). We found significantly higher levels of portal endotoxin in the CA-CPR group compared to the CON (p=0.026) and the CA group (p=0.026). The number of positive mesenteric lymph nodes cultures for *E. coli* was greater in the CA-CPR group, followed by the CA and CON groups, although the difference was not significant (67%, 33%, and 33%, respectively; p=0.407).

Conclusions

Malondialdehyde content and portal endotoxin levels do not increase during the cardiac arrest interval, but only after CPR and ROSC. Although the number of positive MLNs cultures was greater in the CA-CPR animals, no statistically significant differences were observed between the three groups due to the short monitoring period.

## Introduction

Cardiac arrest is a medical emergency with more than one million cases per year and dismal prognosis. Even if return of spontaneous circulation (ROSC) is achieved, survival to hospital discharge ranges from 9.4% for out-of-hospital cardiac arrest (OHCA) to 22.4% for in-hospital cardiac arrest [1-3]. In addition, the risk for post-resuscitation septic complications is high; more than one-third of OHCA victims become bacteremic within the first 12 hours after ROSC, while bacteraemia or other confirmed source of infection may aggravate neurologic outcomes and increase mortality [4].

Bacterial translocation (BT) is the passage of viable bacteria or endotoxins from the gastrointestinal lumen, through the mucosal epithelium, to extra-luminal tissues, such as the mesenteric lymph nodes (MLNs) and other distant organs [5-7]. This phenomenon is usually observed after intestinal ischemia-reperfusion (I/R) injury, but the timing and the associated pathophysiological mechanisms have been poorly investigated after cardiac arrest and resuscitation.

The primary aim of this study was to investigate when BT occurs after the onset of cardiac arrest in animals anaesthetized with propofol-based total intravenous anaesthesia. Secondary aims were the assessment of intestinal oxidative stress and intestinal injury post-cardiac arrest.

## Materials and methods

The study protocol was approved by the Greek General Directorate of Veterinary Services (license No 4978/16-10-2015) and was conducted in accordance to the Greek legislation regarding ethical and experimental procedures.

Animal preparation

Study subjects were 18 female Landrace/Large-White piglets, aged 10-15 weeks, with an average weight of 19±2kg. The animals were purchased from the same breeder (Validakis, Koropi, Greece) and were transported to the research facility (Experimental Research Center, ELPEN, European Ref No. EL 09 BIO 03) one week before experimentation. Prior to the experimental procedure, the animals were fasted overnight but had free access to water [8]. All animals were examined by a veterinarian on the day of the experimentation and were found healthy.

Animals were premedicated with intramuscular injections of 10 mg/kg ketamine hydrochloride (Merial, Lyon, France), 0.5 mg/kg midazolam (Roche, Athens, Greece), and 0.05 mg/kg atropine sulphate (Demo, Athens, Greece), as previously described [9]. The animals were subsequently transported to the operation research facility and intravascular access through the auricular veins was obtained. Anaesthesia was induced by an intravenous bolus dose of 2 mg/kg propofol (Diprivan 1% w/v; Astra Zeneca, Luton, United Kingdom) and 2 μg/kg fentanyl (Janssen Pharmaceutica, Beerse, Belgium) [10]. Whilst spontaneously breathing but anaesthetized, the animals were intubated with an endotracheal tube (Portex, 4.5 mm ID; Mallinckrodt Medical, Athlone, Ireland) and were immobilized in the supine position on a surgical table. Additional 1 mg/kg propofol, 0.15 mg/kg cis-atracurium (Nimbex 2 mg/mL; GlaxoSmithKline, Athens, Greece), and 0.01 mg/kg fentanyl were administered to achieve synchrony with the ventilator. The animals were ventilated with a tidal volume of 10 ml/kg and 21% oxygen (Alpha Delta lung ventilator, Siare, Bologna, Italy), while propofol 0.1 mg/kg/min, cis-atracurium 20 μg/kg/min, and fentanyl 0.6 μg/kg/min were administered to maintain adequate anaesthetic depth. End-tidal carbon dioxide pressure (ETCO2) was continuously monitored (Tonocap-TC200; Datex Engstrom, Helsinki, Finland) and the respiratory rate was adjusted to maintain ETCO2 between 35 and 45 mmHg. Pulse oximetry was monitored throughout the experiment. Electrocardiographic monitoring included leads I, II, III, aVR, aVL, and aVF. The leads were connected to a monitor (Mennen Medical, Envoy; Papapostolou, Athens, Greece) which electronically calculated the heart rate. Arterial blood gases were measured on a blood-gas analyzer (IRMA SL Blood Analysis System, part 436301; Diametrics Medical Inc, Roseville, MN).

For measurement of the aortic pressures, an arterial catheter (model 6523, USCI CR, Bart; Papapostolou) was inserted and forwarded into the descending aorta after surgical preparation of the right internal carotid artery. The systolic (SAP) and diastolic (DAP) arterial pressures were recorded, whereas mean arterial pressure (MAP) was determined by the electronic integration of the aortic blood pressure waveform. The right internal jugular vein was cannulated with a catheter to measure central venous pressure (CVP). The left internal jugular vein was also surgically prepared and a catheter was inserted for fluid administration. Intravascular catheters were attached to pressure transducers that were aligned to the level of the right atrium and were calibrated before their use. This allowed the recording of CVP, right atrial, and arterial pressures. Cardiac output (CO) was measured as the product of time-velocity integral of Doppler transaortic flow, the diameter of the aortic valve, and heart rate, while SVR was calculated using the formula SVR = (MAP - CVP)/CO x 80, as previously described [11].

Experimental procedure

Baseline data were collected after allowing each animal to stabilize for a 30-minute period. Before the experimental procedure, the animals were randomly divided using closed envelopes into three groups: control (CON, n=6), cardiac arrest (CA, n=6) and cardiac arrest and CPR (CA-CPR, n=6). We used three groups to investigate the evolution of intestinal damage during the different stages of cardiac arrest and resuscitation. The investigators involved in data recording, data entry, and data analysis were blinded to each animal's allocation.

In the CON group, the animals were subjected only to minor aseptic procedures (instrumentation) and were monitored for two hours. In the CA and CA-CPR group, a 5F flow-directed pacing catheter (Pacel, 100 cm; St Jude Medical, Ladakis, Athens, Greece) was advanced into the apex of the right ventricle and ventricular fibrillation was induced using a 9V ordinary cadmium battery, as previously described [12]. Cardiac arrest was recognized electrocardiographically and confirmed by loss of arterial pulse. Mechanical ventilation and administration of anaesthetics were discontinued simultaneously with the onset of cardiac arrest and the animals were left untreated for eight minutes (representing the average time of EMS arrival in Europe).

In the CA group, the animals were not resuscitated. Blood samples were collected immediately after the eighth minute of cardiac arrest and then the animals underwent necropsy. Tissue samples from MLNs, liver, spleen, and lungs were also collected. In the CA-CPR group, resuscitation was immediately started after the eighth minute of cardiac arrest according to the 2015 European Resuscitation Council guidelines on resuscitation, with ventilation in 100% oxygen and chest compressions at a rate of 100/min (LUCAS 2 CPR device, Jolife, Lund, Sweden) [13]. Defibrillation was attempted with a 4 J/kg monophasic waveform shock delivered between the right infraclavicular area and the cardiac apex (Primedic Defi-B Defibrillator; Metrax GmbH, Rottweil, Germany), while adrenaline was administered at a dose of 0.02 mg/kg via the marginal auricular vein [8]. Resuscitation efforts continued until ROSC (MAP of at least 60 mm Hg for a minimum of five minutes) or when asystole occurred after the tenth minute of CPR. After ROSC, the animals were anaesthetized and monitored for two hours, during which no further resuscitation was attempted.

Subsequently, anaesthesia was discontinued in the CON and CA-CPR groups, all catheters were removed as previously described, and manual ventilation was initiated [14]. Atropine 0.2 mg/kg followed by neostigmine 0.05 mg/kg were administered after detection of spontaneous swallowing reflex and extubation was performed when adequate inspiration depth was confirmed. Each animal was then transferred to its house for observation. After 24 hours, the animals were humanely euthanized by an intravenous overdose of pentobarbital and underwent necropsy [15]. Blood and tissue samples were also collected.

Malondialdehyde and endotoxin analysis - Histopathological evaluation

Using aseptic techniques, tissue samples of 2.0-3.0 cm from terminal ileum and colon were collected and stored in liquid nitrogen at -70 °C until analysis. Additional samples of ileum and colon were collected and immediately stored in 10% formaldehyde. Blood samples from the portal vein and the aorta were collected and immediately stored in endotoxin-free tubes (EndoGrade® Glass Test Tubes, Hyglos/bioMérieux, Bernried, Germany) until analysis. Plasma levels of endotoxin in the portal and the systemic circulation were determined using a commercially available ELISA kit (KIT LS-F15272, Life Span BioSciences, Inc., Seattle, WA, USA). Tissue malondialdehyde (MDA) content in homogenized ileum and colon was measured using a commercially available ELISA kit (KIT EU2577, Wuhan Fine Biological Technology Co., Ltd., Wuhan, China). The degree of intestinal tissue injury was graded from 0 to 5, using a modified Chiu’s method, as previously described [16].

Statistical analysis

During study design, the insufficient data in literature did not allow for power analysis and sample size estimation. Therefore, we were given permission to conduct a pilot study with six animals per group. The pilot data analysis revealed an adequate level of statistical significance and considering the guiding principles underpinning the humane use of animals in scientific research (three Rs), the final sample size included six animals per group [15,17]. Data were expressed as mean±SD or median±IQR for continuous variables. The normality of distributions was assessed using the Kolmogorov-Smirnov test. Categorical variables were compared between groups using the chi-square test, or Fisher's exact test, as required. Comparisons of continuous variables between the three groups were performed using one-way ANOVA and pairwise comparisons were performed using the Bonferroni test. In case of non-normal distributions, the non-parametric Kruskal-Wallis and Mann-Whitney tests were used. Comparisons of continuous data extracted at the predefined time points of the observation period, only for the CON and CA-CPR groups, were performed using independent samples t-test, or Mann-Whitney test in case of violation of normality. All tests were two-tailed and a value of p<0.05 was considered significant. Statistical analysis was performed using SPSS version 21.0 (IBM Corp, Armonk, NY, USA).

## Results

All animals in the CA-CPR group were successfully resuscitated.

Haemodynamic and metabolic parameters

No statistically significant differences were observed in baseline haemodynamic and metabolic parameters between the three groups (Table 1).

**Table 1:** Baseline hemodynamic and metabolic parameters of the study CON, control group; CA, cardiac arrest group; CA-CPR, cardiac arrest and cardiopulmonary resuscitation group.

Measurement	CON	CA	CA-CPR	p-value
	Mean±SD	Mean±SD	Mean±SD	
Heart rate (beats/min)	106.83 ± 11.53	107.00 ± 5.73	96.33 ± 14.99	0.212
Systolic aortic pressure (mmHg)	115.50 ± 7.4	116.83 ± 10.34	115.17 ± 10.74	0.951
Diastolic aortic pressure (mmHg)	92.17 ± 5.78	94.00 ± 10.86	89.67 ± 10.09	0.720
Systolic right atrial pressure (mmHg)	12.00 ± 4.43	10.17 ± 3.06	13.50 ± 3.02	0.297
Diastolic right atrial pressure (mmHg)	6.83 ± 2.48	5.33 ± 2.42	8.33 ± 2.50	0.144
Cardiac output (L/min)	5.53 ± 0.61	4.93 ± 1.00	4.90 ± 0.99	0.399
pH	7.44 ± 0.05	7.39 ± 0.03	7.41 ± 0.04	0.160
pO_2_ (mmHg)	128.17 ± 19.05	111.33 ± 17.37	119.00 ± 16.75	0.287
pCO_2_ (mmHg)	42.5 ± 4.04	45.83 ± 2.40	42.67 ± 4.27	0.240
HCO_3_ (mmol/L)	27.68 ± 2.87	26.50 ± 1.63	26.20 ± 2.25	0.515
Lactate (mmol/L)	0.51 ± 0.32	0.58 ± 0.15	0.97 ± 0.91	0.348

All animals in the CA-CPR group restored ROSC after 6.3±2.1 min of CPR and a mean adrenaline dose of 0.5±0.17.

Statistically significant differences were observed in pH (p=0.002), HCO3 (p=0.003) and lactate (p=0.008) between the CON and the CA-CPR group one hour after ROSC. Two hours after ROSC, pH and HCO3 levels remained significantly lower (p=0.016 and p=0.007, respectively), whereas lactate concentration remained significantly higher (p=0.046) in the CA-CPR group (Table 2).

**Table 2:** Hemodynamic and metabolic parameters in the CON and CA-CPR groups during the monitoring period CON: control group; CA: cardiac arrest group; CA-CPR: cardiac arrest and cardiopulmonary resuscitation group.

Measurement	1 hour			2 hours		
	CON	CA-CPR	p-value	CON	CA-CPR	p-value
	Mean±SD	Mean±SD		Mean±SD	Mean±SD	
Heart rate (beats/min)	101.67 ± 9.50	110.50 ±13.20	0.213	99.83 ± 8.26	103.17 ± 12.40	0.596
Systolic aortic pressure (mmHg)	112.83 ± 13.4438	103.17 ±7.76	0.158	112.00 ± 18.49	117.50 ± 7.77	0.524
Diastolic aortic pressure (mmHg)	86.17 ± 10.70	82.50 ± 6.66	0.492	82.33 ± 16.37	93.83 ± 7.94	0.164
Systolic right atrial pressure (mmHg)	11.50 ± 2.43	13.17 ± 2.79	0.295	12.17 ± 2.56	13.50 ± 3.51	0.469
Diastolic right atrial pressure (mmHg)	7.67 ± 2.58	8.17 ± 2.48	0.740	7.83 ± 2.40	8.33 ± 3.39	0.774
Cardiac output (L/min)	6.05 ± 1.48	5.55 ± 0.82	0.485	6.25 ± 1.41	4.83 ± 0.79	0.064
pH	7.4 ± 0.05	7.28 ± 0.06	0.002	7.41 ± 0.05	7.32 ± 0.06	0.016
pO_2_(mmHg)	128.17 ± 27.86	108.33 ±19.75	0.185	121.17 ± 29.67	113.17 ± 28.43	0.644
pCO_2_(mmHg)	43.17 ± 4.40	40.50 ± 2.59	0.229	42.33 ± 3.56	39.50 ± 2.26	0.131
HCO_3_(mmol/L)	26.05 ± 3.94	18.75 ± 2.36	0.003	26.35 ± 3.44	19.78 ± 3.20	0.007
Lactate (mmol/L)	0.70 ± 0.35	3.27 ± 1.85	0.008	0.59 ± 0.47	2.21 ± 1.50	0.046

Intestinal mucosal injury and malondialdehyde content

**Figure 1:**
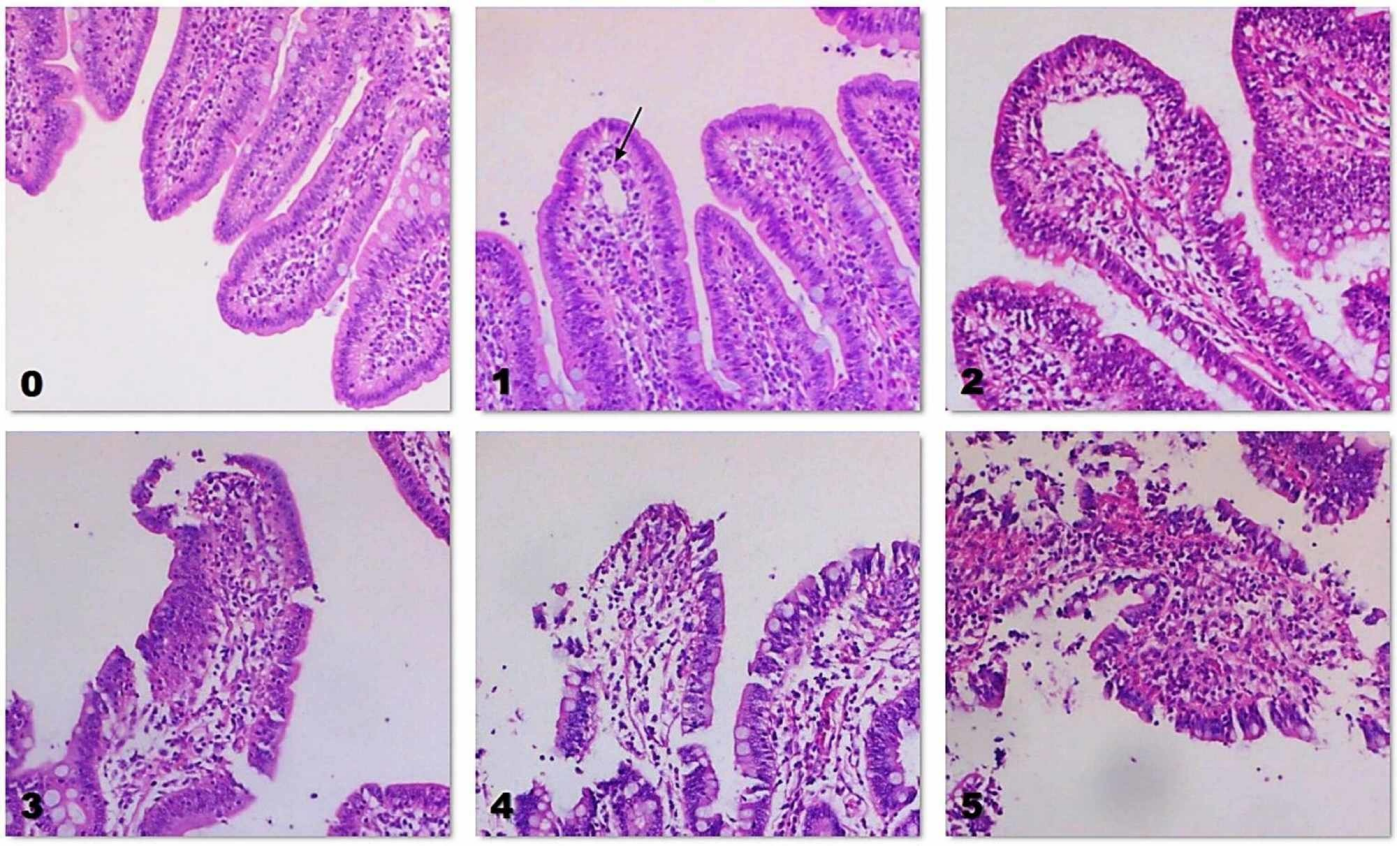
Intestinal mucosal injury in our study. Magnification 200×. 0: Normal villi – CON group. 1: Development of the sub-epithelial Gruenhagen’s space (black arrow) with some capillary congestion – CON group. 2: Extension of the sub-epithelial space with moderate lifting of the epithelial layer from the lamina propria – CA group. 3: Massive epithelial lifting with a few denuded villi – CA group 4: Denuded villi with exposure of dilatated capillaries – CA-CPR group. 5: Digestion and disintegration of lamina propria, hemorrhage and ulceration – CA-CPR group. CON: Control; CA: cardiac arrest; CA-CPR: cardiac arrest-cardiopulmonary resuscitation

Although histological analysis revealed mild intestinal damage in the CA and CA-CPR groups, there was no statistically significant difference in intestinal mucosal injury score between the three groups (p=0.504) (Table 3, Fig. 1).

**Table 3:** Comparison of malondialdehyde content and mucosal injury between the three groups ^a^ p=0.016 vs CA; ^b^ p=0.004 vs CA-CPR; ^c^ p=0.016 vs CA-CPR MDA: malondialdehyde; CON: control group; CA: cardiac arrest group; CA-CPR: cardiac arrest and cardiopulmonary resuscitation group.

	CON		CA		CA-CPR		p-value
	Median	IQR	Median	IQR	Median	IQR	
MDA – Ileum (ng/ml)	23.02	4.51	20.99	7.77	27.46^a^	4.53	0.042
MDA – Colon (ng/ml)	9.73^b^	8.06	10.56^ c^	8.19	18.82	10.36	0.006
Mucosal Injury Score	2.00	2.50	3.00	2.50	3.00	1.00	0.504

**Figure 2:**
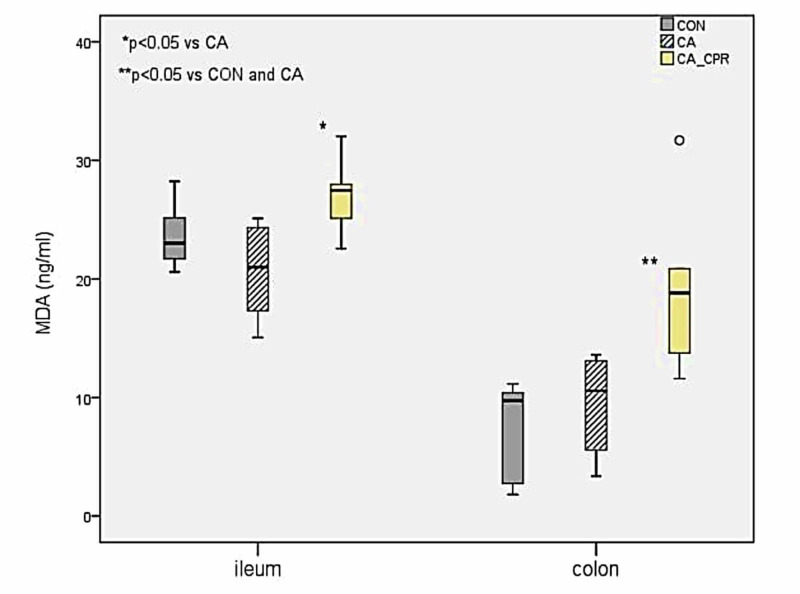
Malondialdehyde concentration in homogenized ileum-colon. CON: control group; CA: cardiac arrest group; CA-CPR: cardiac arrest and cardiopulmonary resuscitation group.

We found no statistically significant difference in the MDA content of the homogenized ileum (p=0.337) and colon (p=0.337) between the CA and CON groups. Malondialdehyde content in homogenized ileum was significantly higher in the CA-CPR group compared to the CA group (p=0.016), but not compared to the CON group (p=0.109). Also, MDA measurements in homogenized colon revealed significantly higher levels in the CA-CPR group compared to the CON (p=0.004) and the CA group (p=0.016) (Figure 2).

Endotoxin levels

**Figure 3:**
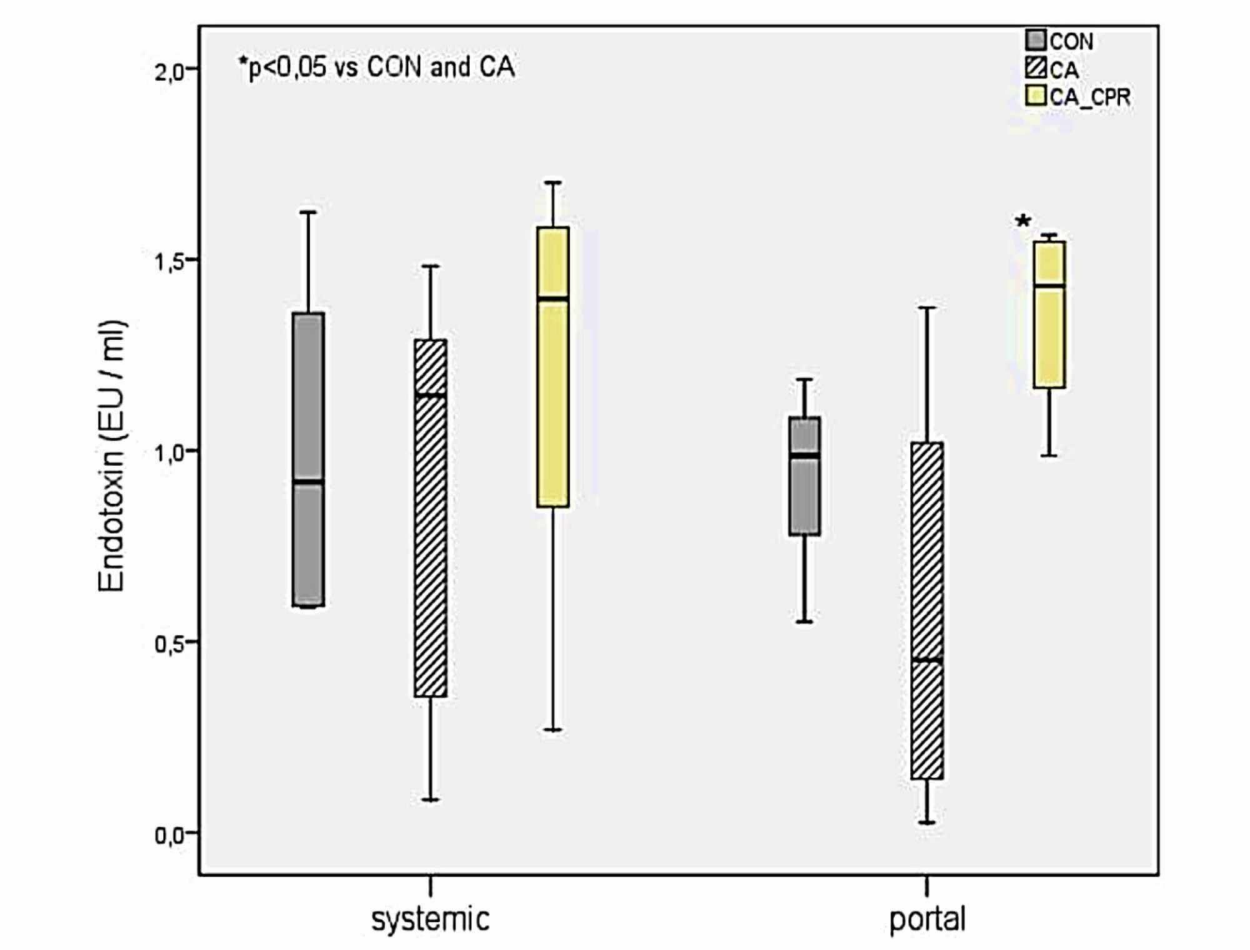
Endotoxin levels in systemic and portal circulation. CON: control group; CA: cardiac arrest group; CA-CPR: cardiac arrest and cardiopulmonary resuscitation group.

No statistically significant differences were observed in endotoxin levels in the systemic circulation between the three groups (p=0.628). On the contrary, measurements in the portal circulation revealed significantly higher levels of endotoxin in the CA-CPR group compared to the CA (p=0.026) and the CON (p=0.026) group. However, there was no significant difference in portal endotoxin levels between the CA and the CON group (p=0.240) (Figure 3).

Blood and tissue cultures

The number of positive MLNs cultures for *E. coli *was greater in the CA-CPR group, followed by the CA and CON groups, although the difference was not significant (67%, 33%, and 33%, respectively; p=0.407). All other blood and tissue cultures collected at 24 hours post-ROSC were not indicative of BT.

## Discussion

Despite advances in resuscitation science and the increasing evidence on the pathophysiology of post-resuscitation syndrome, the time at which BT occurs after cardiac arrest remains unknown. In this study, we found that CA-CPR animals had significantly higher levels of endotoxin in portal circulation compared to CA and CON animals. In addition, the CA-CPR animals had more positive MLNs cultures compared to the other groups, although the difference was not statistically significant. To our knowledge, this is the first study presenting direct evidence regarding the timing of BT after cardiac arrest.

Malondialdehyde, the end product of fatty acid peroxidation, is a good indicator of oxidative injury and the degree of lipid peroxidation and oxidative stress [18]. In our study, we did not find significant differences in the MDA content of the homogenized ileum and colon between the CA and CON groups. Considering that our animals were healthy without any comorbidity, we conclude that intestinal ischaemia alone could not increase oxidative stress during the eight-minute cardiac arrest interval, indicating significant intestinal physiological reserves possibly due to preserved microcirculatory autoregulation [19]. After the onset of CPR, the gut receives a small amount of the compression-related cardiac output (less than 5%) and thus the major intestinal disturbance during CPR continues to be ischaemia rather than reperfusion [5]. However, even this small amount of oxygenated blood may be capable for inducing reperfusion injury, which is significantly exacerbated after ROSC [20]. This was also evident in the CA-CPR group, in which intestinal reperfusion increased inflammation and the oxidative stress, resulting in a higher MDA content in these animals.

Despite the significant differences in the MDA content of our animals, several authors have reported that treatment with propofol decreases intestinal MDA levels [16]. Therefore, propofol may have exerted protection against oxidative stress, resulting in lower MDA levels in study [16]. In another animal study, pre-treatment with a sedative dose of propofol attenuated intestinal epithelial apoptosis after I/R, which was attributed to its antioxidant properties [21]. Similarly, our animals were pre-treated with sedative doses of propofol prior to the induction of cardiac arrest and CPR, which may have significantly alleviated oxidative stress, intestinal injury, and thus BT, suggesting a preconditioning-like effect of propofol [7,16,22]. In addition, it is important to remember that healthy intestinal mucosa expresses low concentrations of toll-like receptor 4 (TLR-4), which is significantly increased in inflammatory conditions [23]. However, propofol has been reported to down-regulate TLR-4 expression, therefore delaying the reperfusion-induced BT [24]. Further studies in different animal models and particularly in the clinical setting are warranted to determine if this response translates to the bedside when propofol is used during the peri-arrest period.

Although research has shown that the reperfused intestine is characterized by severe histological alterations, mucosal rupture, and increased permeability [23], we did not find significant differences in the intestinal mucosal injury score between the three groups. Our results are similar to those of Schroeder et al. who reported a mild intestinal barrier damage and mild local intestinal inflammation within 24 h post-ROSC in a rat model of six-minute cardiac arrest [25]. The mild intestinal injury in the CA-CPR group may be also explained by the low dose of the administered adrenaline, which may have minimized the detrimental effect of the drug on mesenteric blood flow [26]. Also, the non-significant differences in endotoxin levels between the CA and the CON group imply that the ischaemic-induced intestinal injury was not able to cause BT. On the other hand, reperfusion of the intestine after CPR and ROSC induced BT, significantly increasing endotoxin levels in the portal circulation of the CA-CPR group [27]. Of note, research has shown that endotoxin increases in more than 24 hours after ROSC, implying that the short monitoring period of 24 hours may be the reason for the non-significant differences in systemic endotoxin levels in our study [27]. Another explanation for this finding may be the circulation of endotoxin, which either translocates through the portal venous system to the liver or enters the systemic circulation via the thoracic duct. Then, a significant fraction of endotoxin binds to plasma proteins and is removed by certain organs, particularly the liver [28].

Although our findings imply that reperfusion may play a causative role in intestinal injury, possibly due to overproduction of oxygen radicals and/or insufficient antioxidant activity, the difference in MDA concentration in ileum and colon may be inversely correlated with the amount of bacteria species and populations commonly hosted in these two sites of the gut. However, gut-derived BT may not be the sole mechanism of post-resuscitation inflammation and sepsis. It is possible that bacteria, macromolecules, immune cells, and cytokines travel along the mesenteric lymphatics to the systemic circulation, entering pulmonary circulation and inducing acute lung injury and multiple organ dysfunction [29]. As the gut-portal and the gut-lymph mechanism may occur independently of each other, further studies are required for the elucidation of these phenomena.

Limitations to our study should be considered. First, our experiment was conducted on apparently healthy pigs with no underlying disease. This is not the case in human cardiac arrest victims who, most of the time, have various comorbidities. Second, the monitoring period of the CA-CPR group was limited to 24 hours and different results could be obtained in longer monitoring periods. Due to the small sample size of our study, statistical analysis may have failed to capture other factors associated with BT. Moreover, we did not search for TLR-4 polymorphisms, although some of them may prevent the deleterious effect of endotoxin [30]. In addition, we did not perform repeated measurements of endotoxin. Nevertheless, all measurements were performed at 24 hours following ROSC.

## Conclusions

This is the first study presenting direct evidence regarding the timing of BT after cardiac arrest. Malondialdehyde content and portal endotoxin levels increased significantly only after CPR and ROSC. Although the number of positive MLNs cultures was greater in the CA-CPR animals, no statistically significant differences were observed between the three groups at 24 hours. Future studies on BT should include a post-reperfusion monitoring period of more than 48 hours.
